# Inhibition of H3K9me2 Reduces Hair Cell Regeneration after Hair Cell Loss in the Zebrafish Lateral Line by Down-Regulating the Wnt and Fgf Signaling Pathways

**DOI:** 10.3389/fnmol.2016.00039

**Published:** 2016-05-26

**Authors:** Dongmei Tang, Qin Lin, Yingzi He, Renjie Chai, Huawei Li

**Affiliations:** ^1^Department of Otorhinolaryngology, Affiliated Eye and ENT Hospital of Fudan UniversityShanghai, China; ^2^Department of Otolaryngology Head and Neck Surgery, First Affiliated Hospital of Fujian Medical UniversityFuzhou, China; ^3^Key Laboratory for Developmental Genes and Human Disease, Ministry of Education, Institute of Life Sciences, Southeast UniversityNanjing, China; ^4^Co-innovation Center of Neuroregeneration, Nantong UniversityNantong, China; ^5^State Key Laboratory of Medical Neurobiology, Fudan UniversityShanghai, China; ^6^Institute of Stem Cell and Regeneration Medicine, Institutions of Biomedical Science, Fudan UniversityShanghai, China; ^7^Key Laboratory of Hearing Science, Ministry of Health, EENT Hospital, Fudan UniversityShanghai, China

**Keywords:** H3K9me2, hair cell regeneration, zebrafish, Fgf signaling pathway, Wnt signaling pathway

## Abstract

The activation of neuromast (NM) supporting cell (SC) proliferation leads to hair cell (HC) regeneration in the zebrafish lateral line. Epigenetic mechanisms have been reported that regulate HC regeneration in the zebrafish lateral line, but the role of H3K9me2 in HC regeneration after HC loss remains poorly understood. In this study, we focused on the role of H3K9me2 in HC regeneration following neomycin-induced HC loss. To investigate the effects of H3K9me2 in HC regeneration, we took advantage of the G9a/GLP-specific inhibitor BIX01294 that significantly reduces the dimethylation of H3K9. We found that BIX01294 significantly reduced HC regeneration after neomycin-induced HC loss in the zebrafish lateral line. BIX01294 also significantly reduced the proliferation of NM cells and led to fewer SCs in the lateral line. *In situ* hybridization showed that BIX01294 significantly down-regulated the Wnt and Fgf signaling pathways, which resulted in reduced SC proliferation and HC regeneration in the NMs of the lateral line. Altogether, our results suggest that down-regulation of H3K9me2 significantly decreases HC regeneration after neomycin-induced HC loss through inactivation of the Wnt/β-catenin and Fgf signaling pathways. Thus H3K9me2 plays a critical role in HC regeneration.

## Introduction

Hearing loss and balance disorders in humans are mainly caused by sensory hair cell (HC) loss. The inner ear sensory HCs are highly metabolic and are sensitive to a wide variety of noxious insults, and the etiology of most human hearing loss is loss of HCs from ototoxic drugs, noise exposure, trauma, disease, aging, and genetic disorders (Mills and Going, 1982; Henley and Rybak, 1995). HCs do not normally regenerate in adult mammals (Roberson and Rubel, 1994), thus hearing loss caused by HC loss is irreversible in humans. However, several studies have reported that a limited amount of HC regeneration can occur in the vestibular sensory epithelium and in early postnatal mammalian cochleae (Forge et al., 1998; Burns et al., 2012; Cox et al., 2014). In contrast, HCs in non-mammalian vertebrates are regenerated in both auditory and vestibular systems after HC loss, and this leads to functional recovery of hearing and balance function (Corwin and Cotanche, 1988; Ryals and Rubel, 1988). Thus, further understanding of the mechanisms of HC regeneration in non-mammalian vertebrates might shed light on new therapeutic targets for restoring hearing and balance in humans.

The zebrafish lateral line system consists of a group of rosette-like sensory organs named neuromasts (NMs) that are located on the head and along the body of the zebrafish in a species-specific pattern (Metcalfe et al., 1985; Ledent, 2002). The mature lateral line NM consists of HCs in the center and SCs surrounding the HCs. The HCs in the zebrafish lateral line share functional and structural similarities with the HCs in the mammalian inner ear (Raible and Kruse, 2000; Nicolson, 2005), and the surface location of the neuromasts makes the HCs susceptible to ototoxic insults such as aminoglycoside antibiotics and chemotherapy agents in a similar manner to their counterparts in mammalian and avian inner ears (Harris et al., 2003; Giari et al., 2012). Because zebrafish have the capacity to spontaneously regenerate lost HCs after they have been damaged, the zebrafish lateral line system offers a convenient and efficient model for studying HC regeneration (Brignull et al., 2009). Several studies have shown that SCs in the sensory epithelium of the inner ear are sources for HC regeneration after HC loss, and the newly regenerated HCs usually arise through the proliferation and differentiation of SCs, which is known as mitotic regeneration (Corwin and Cotanche, 1988; Fekete et al., 1998). Alternatively, SCs also have the ability to directly trans-differentiate into new HCs without cell cycle re-entry (Roberson et al., 2004; Hernández et al., 2007).

Epigenetic modifications play key roles in the regulation of many chromosomal functions and are involved in and closely related to many biological events, including the regulation of transcription; the survival, differentiation, and apoptosis of cells; and the regeneration of tissues (Rugg-Gunn et al., 2010; Greer and Shi, 2012; Yu et al., 2013). Chromatin remodeling is an important epigenetic modification process, and in most cases chromatin remodeling is achieved by post-translational modification of the histone amino terminal tails, including acetylation, methylation, phosphorylation, and ubiquitination (Berger, 2002). Histone methylation and demethylation are the major covalent histone modifications that have been linked to the regulation of gene transcription. Histone methylation is catalyzed by the histone methyltransferases, while the removal of methyl groups from histone lysine residues is catalyzed by the histone demethylases (Agger et al., 2008). Recent studies have demonstrated the specific function of histone methyltransferases/demethylases in many cellular processes such as cell cycle regulation and cellular differentiation, survival, death, and proliferation (Shi, 2007; Yoshimi and Kurokawa, 2011). However, the role of histone methylation/demethylation in supporting cell (SC) proliferation and HC regeneration remains largely unknown.

Dimethylation of lysine 9 of histone H3 (H3K9me2) is a dynamic histone methylation marker that functions in euchromatin gene silencing, and changes in H3K9me2 levels are implicated in both carcinogenesis and embryogenesis (Zernicka-Goetz et al., 2009; Stark et al., 2011). BIX01294 is a G9a and G9a-like protein (GLP) inhibitor that efficiently inhibits the G9a/GLP-mediated dimethylation of H3K9 and has been used to investigate the functions of H3K9me2 (Kubicek et al., 2007; Chang et al., 2009). It has been reported that BIX01294 suppresses cell proliferation, migration, and invasion in certain types of rapidly proliferating cancer cells (Ma et al., 2010; Varier and Timmers, 2011), but the effects of H3K9me2 in regulating SC proliferation and HC regeneration after HC loss remain unknown.

Our previous study reported that pharmacological inhibition of G9a/GLP with BIX01294 significantly decreases H3K9me2 levels and prevents neomycin-induced ototoxicity and HC apoptosis in mouse cochlear epithelium (Yu et al., 2013). However, whether BIX01294 treatment has any effect on SC proliferation and HC regeneration after HC loss remains unknown in zebrafish. In this study, we examined the effect of BIX01294 treatment in HC regeneration after neomycin-induced HC loss in the zebrafish lateral line and investigated the possible mechanisms involved in this effect. We found that inhibition of G9a/GLP with BIX01294 after neomycin treatment significantly suppressed cell proliferation and decreased HC regeneration in zebrafish neuromasts. Moreover, we found that BIX01294 treatment after neomycin damage significantly down-regulated the expression of target genes of the Wnt/β-catenin and Fgf activation. All of these results suggest that inhibition of H3K9me2 reduces cell proliferation and HC regeneration in the zebrafish lateral line through down-regulation of the Wnt and Fgf signaling pathways.

## Materials and Methods

### Zebrafish Maintenance

Both adult wild-type and Tg(brn3c:mGFP)^s356t^ transgenic zebrafish and their spawning embryos were raised in embryo medium (EM) at 28.5°C according to standard protocols (Detrich et al., 1999). Ages of embryos are given as days post-fertilization (dpf). The Animal Care and Use Committee of Fudan University approved for all of the zebrafish experiments.

### Neomycin Treatment and Pharmacological Administration

Neomycin sulfate (Sigma-Aldrich) was added at a final concentration of 400 μM, and the 5 dpf larvae were incubated for 1 h at 28.5°C. The larvae were allowed to recover at 28.5°C followed by three rinses in fresh EM. The H3K9 dimethylation inhibitors BIX01294 (Sigma-Aldrich) and UNC0638 (Sigma-Aldrich) were dissolved in DMSO both at stock concentrations of 10 mM and then diluted to the final concentrations in fresh EM. The neomycin-damaged larvae were incubated with medium containing BIX01294 (2, 5, 10 or 20 μM) or UNC0638 (10 μM) as the experimental groups, and the same concentrations of DMSO were used as controls. BIX01294, UNC0638, or DMSO was added to the larvae immediately after the neomycin was washed away, and the larvae were incubated at 28.5°C for 24 h or 48 h prior to analysis.

### Cell Proliferation Assay

BrdU (Sigma) was used to label the proliferating cells. After 1 h neomycin treatment, the larvae were incubated in fresh EM with 10 mM BrdU for 24 h or 48 h at 28.5°C.

### HC Labeling and Immunohistochemistry

FM1-43FX dye (Molecular Probes) was used to observe and image the functional HCs within the neuromasts. FM1-43FX dye can enter the mature HCs through mechanotransduction channels. FM1-43FX dye at a final concentration of 3 μM was added to live 5 dpf larvae for 45 s and then rinsed three times with fresh water. The larvae were anesthetized in 0.02% MS-222 and fixed with 4% PFA overnight at 4°C.

For immunohistochemistry analysis, the fixed larvae were washed several times with PBT-2 (PBS containing 0.5% Triton X-100) for 30 min at room temperature then incubated in blocking solution for 1 h at 37°C. The primary antibodies were anti-Myosin-VI (1:200 dilution; Proteus BioSciences), anti-GFP (green fluorescent protein; 1:1000 dilution; Abcam), anti-dimethyl H3K9 (1:1000 dilution; Abcam), anti-Sox2 (1:200 dilution; Abcam), and anti-cleaved caspase-3 (1:500 dilution; Cell Signaling Technology Inc., Danvers, MA, USA). The larvae were incubated with primary antibodies overnight at 4°C and then washed three times with PBT-2 and incubated with secondary antibodies (1:200 dilution; Invitrogen) for 1 h at 37°C. The larvae were then incubated with 4,6-diamidino-2-phenylindole (DAPI; Invitrogen) for 20 min at room temperature to label the nuclei.

Immunohistochemistry staining was also used to detect the BrdU incorporation. To denature the DNA before antibody staining, the fixed larvae were washed three times with PBT-2 and then treated with 2 N HCl for 30 min at 37°C. The larvae were then incubated in blocking solution (PBT-2 with 10% normal donkey serum) for 1 h at 37°C and then incubated with anti-BrdU antibody (1:200 dilution; Santa Cruz Biotechnology) overnight at 4°C. The larvae were washed three times with PBT-2 and incubated with secondary antibody (1:200 dilution; Invitrogen) for 1 h at 37°C, and specimens were examined with a Leica confocal fluorescence microscope (TCS SP8; Leica).

### Western Blot Analysis

Total protein was isolated from each of 10 larvae with the AllPrep DNA/RNA/Protein Mini Kit (QIAGEN, Hilden, Germany) according to the manufacturer’s instructions. The protein concentrations were determined using a bicinchoninic acid (BCA) protein kit (Thermo, Fisher Scientific, Rockford, IL, USA). The proteins were separated on sodium dodecyl sulfate (SDS)-polyacrylamide gels and transferred onto polyvinylidene difluoride (PVDF) membranes (Immobilon-P; Millipore, Bedford, MA, USA) after electrophoresis. The membranes were blocked with 5% non-fat dried milk in Tris-buffered saline with Tween 20 (TBST; 50 mM Tris-HCl (pH 7.4), 150 mM NaCl, and 0.1% Tween-20) for 1 h at room temperature and then blotted overnight with anti-dimethyl H3K9 (1:1000 dilution; Abcam) at 4°C with β-actin as control. After rinsing three times (10 min each) with TBST, the membranes were incubated with secondary antibody (1:1000 dilution; Invitrogen) for 1 h at room temperature. The immunoreactive bands were visualized using an ECL kit (Pierce). To quantify the relative levels of the protein, the intensities of the bands were quantified with Image J.

### Cell Counts and Statistical Analysis

Cells in the first five posterior lateral line neuromasts (L1–L5) were counted. All statistical analyses were performed with IBM SPSS (version 21 for Mac) and GraphPad Prism (version 6 for Mac). Data were analyzed using Student’s *t*-tests (with two comparisons) or one-way ANOVA (with multiple comparisons). All data are presented as mean ± SEM. *p* < 0.05 was considered statistically significant, and *p* < 0.001 was considered highly significant.

### Whole-Mount *in situ* Hybridization

Regular whole-mount *in situ* hybridization of zebrafish embryos was performed as previously described (Thisse and Thisse, 2008). All primers for probe synthesis are listed in the Table 1. Embryos were mounted in 100% glycerol.

**Table 1:** **Primers used in the study**.

Gene Name	Forward Primer	Reverse Primer
wnt4a	5′-tgcatatggggttgctttctc-3′	5′-gactcttggctatacactcgc-3′
wnt10a	5′-agcgcttctccaaggacttc-3′	5′-gggaatcatggaaacggcag-3′
axin2	5′-accgacaaaccaagcacaag-3′	5′-tccgttttgagttatgaagctct-3′
fgf3	5′-tgagcttcttggatccgagt-3′	5′-tgccgctgactctctctaag-3′
fgf10a	5′-ctgctgcttctgttcctgtg-3′	5′-agtgccttcttctccaaatgg-3′
pea3	5′-agtgtgtttcgtgaaggtgc-3′	5′-atacaagaggatggggtggg-3′
fgfr1	5′-gtatctcgcatccaagaagtgt-3′	5′-agctgtatgtgtttctcccaga-3′
atoh1a	5′-gtcaaagtacgcgagctctg-3′	5′-acttcagtgaggcgagaact-3′

## Results

### The G9a/GLP-Specific Inhibitor BIX01294 Decreases H3K9me2 Levels in Neuromasts

**Figure 1:**
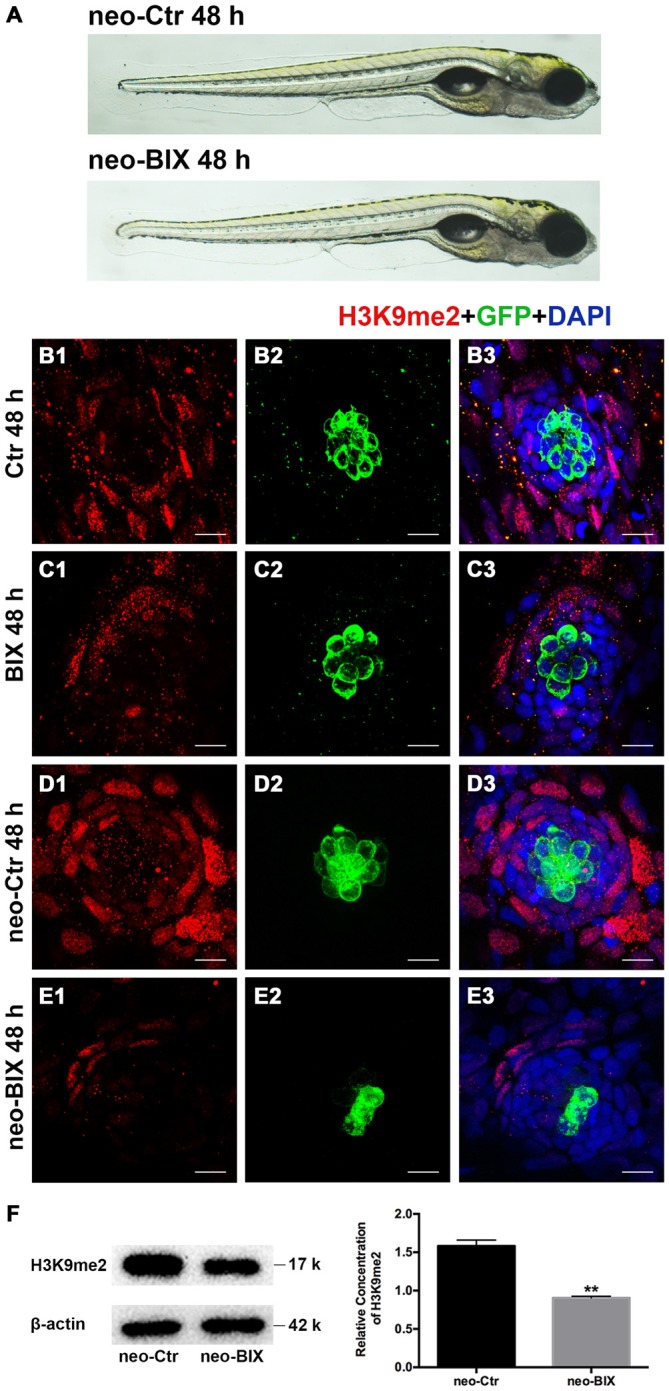
**BIX01294 treatment decreases H3K9me2 levels in zebrafish neuromasts (NMs). (A)** Both the BIX01294 (BIX)-treated group and DMSO control group were morphologically normal following 1 h neomycin treatment. **(B1,D1)** H3K9me2 immunofluorescence (red) is of relatively high intensity in the control zebrafish NMs, and the H3K9me2 level increased at 48 h after neomycin damage for 1 h. **(C1,E1)** BIX01294 treatment reduced the H3K9me2 level, and the intensity of H3K9me2 was significantly decreased with the addition of 20 μM BIX01294 for 48 h following 1 h neomycin damage. **(B2–E3)** Some degree of decreased hair cell (HC; green) regeneration after neomycin treatment for 1 h was also observed after exposure to BIX01294 for 48 h compared to the neomycin treatment controls, while a reduction in HCs was not obvious in the non-neomycin control groups. HCs were labeled with anti-GFP antibody **(B2–C2)** or green fluorescence **(D2–E2)**, and nuclei were labeled with 4,6-diamidino-2-phenylindole (DAPI; blue). Scale bar = 10 μm. **(F)** The reduced H3K9me2 level by 20 μM BIX01294 was reconfirmed with western blotting analysis at 48 h after neomycin damage. β-actin was included as the control. Mean ± SEM for three experimental replicates. **p < 0.01.

We first examined the level of H3K9me2 in neomycin-damaged 5 dpf zebrafish after exposure to 20 μM BIX01294 for 48 h. H3K9me2 immunostaining was performed on transgenic line Tg(brn3c:mGFP)^s356t^ in which differentiated HCs express green fluorescence. Using 20 μM BIX01294 did not increase the death of zebrafish after neomycin damage compared with the controls, and both the 20 μM BIX01294-treated group and the DMSO-treated control group were morphologically normal, suggesting that the larvae did not suffer any drug toxicity during exposure to BIX01294 (Figure 1A). Consistent with previous reports, pharmacological inhibition of G9a/GLP with BIX01294 significantly decreased the H3K9me2 levels in neuromasts compared to controls with or without neomycin damage (Figures 1B–E). Moreover, the significantly decreased H3K9me2 level upon the addition of 20 μM BIX01294 was confirmed by semi-quantitative western blotting analysis (Figure 1F). We also observed fewer HCs when exposed to BIX01294 for 48 h after 1 h neomycin damage in comparison with the neomycin-treated controls (Figures 1D2–E3).

### Inhibition of G9a/GLP Reduces HC Regeneration Ability in the Zebrafish Lateral Line

**Figure 2:**
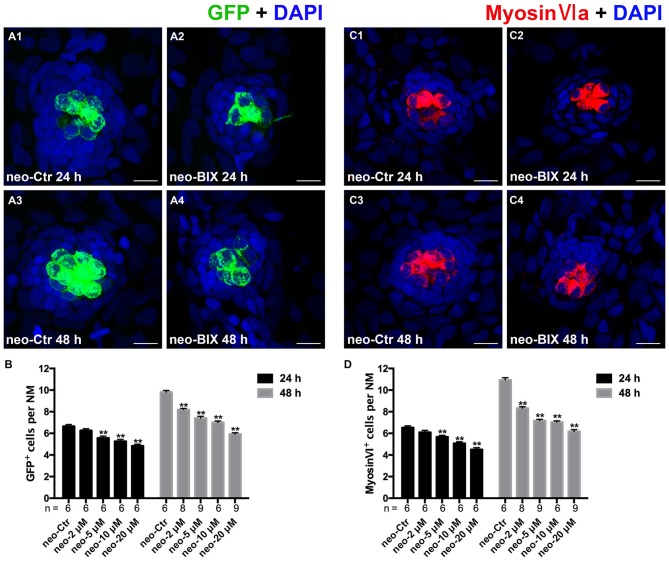
**BIX01294 treatment reduces HC regeneration in the zebrafish lateral line. (A)** We treated 5 days post-fertilization (dpf) Tg (brn3c:mGFP) zebrafish with neomycin for 1 h and then treated them for 24 h or 48 h with BIX01294. HCs in these fish are GFP^+^ (green), and nuclei are stained with DAPI (blue). Scale bars = 10 μm. **(B)** The average number of GFP^+^ cells per NM in larvae treated with or without 20 μM BIX01294 for 24 h or 48 h after neomycin damage for 1 h. Bars are mean ± SEM, and *n* = total number of embryos. **p < 0.001. **(C)** We treated 5 dpf wild-type zebrafish with neomycin for 1 h and then treated them for 24 h or 48 h with BIX01294, and HC-specific Myosin-VI immunostaining (red) was used to label HCs. Nuclei are stained with DAPI (blue). Scale bars = 10 μm. **(D)** The average number of Myosin-VI^+^ cells per NM in larvae treated with or without 20 μM BIX01294 for 24 h or 48 h after neomycin damage for 1 h. Bars are mean ± SEM, and *n* = total number of embryos. **p < 0.001.

To test whether BIX01294 could modulate the proliferation and regeneration of HCs after HC loss, we quantified the changes in the numbers of newly regenerated HCs in the 5 dpf Tg(brn3c:mGFP) zebrafish lateral line 24 h and 48 h after different doses of BIX01294 treatment. Compared with DMSO controls, both the 24 h and 48 h groups treated with BIX01294 showed significant decreases in the number of GFP-positive HCs (Figures 2A1–A4). There was no significant difference between controls and the 2 μM BIX01294-treated group after 24 h; however, after 48 h co-incubation the difference was significant between DMSO controls and the 2 μM BIX01294-treated group (*p* < 0.001). We did not find any significant dose-dependent difference between 5 μM and 10 μM BIX01294 treatment groups after 24 h and 48 h co-incubation, while 20 μM BIX01294 treatment induced fewer GFP-positive HCs in both the 24 h and 48 h groups without increasing cell death compared with controls (*p* < 0.001; Figure 2B).

To further confirm our findings, we also used Myosin-VI immunostaining to specifically label and quantify the HCs of the wild-type larvae. The numbers of HCs in neuromasts L1–L5 were counted in 6–9 fish at both time points. As Figures 2C1–C4, 2C1–C4, D show, treatment with BIX01294 for 24 h after neomycin damage for 1 h resulted in a significant decrease in the number of Myosin-VI^+^ HCs compared to controls (*p* < 0.001), and the decrease was even greater compared to controls at 48 h (*p* < 0.001). Both groups showed dose-dependent decreases except for the 5 μM and 10 μM groups after 48 h treatment. All of these results validated the observation that normal HC regeneration was severely impaired in the presence of the G9a/GLP inhibitor BIX01294. Based on the dose-dependent effect analysis of GFP-positive HCs in the Tg(brn3c:mGFP) zebrafish lateral line and the Myosin-VI staining of HCs in the wild-type larvae, we chose the 20 μM BIX01294 treatment as the experimental group in the following experiments.

**Figure 3:**
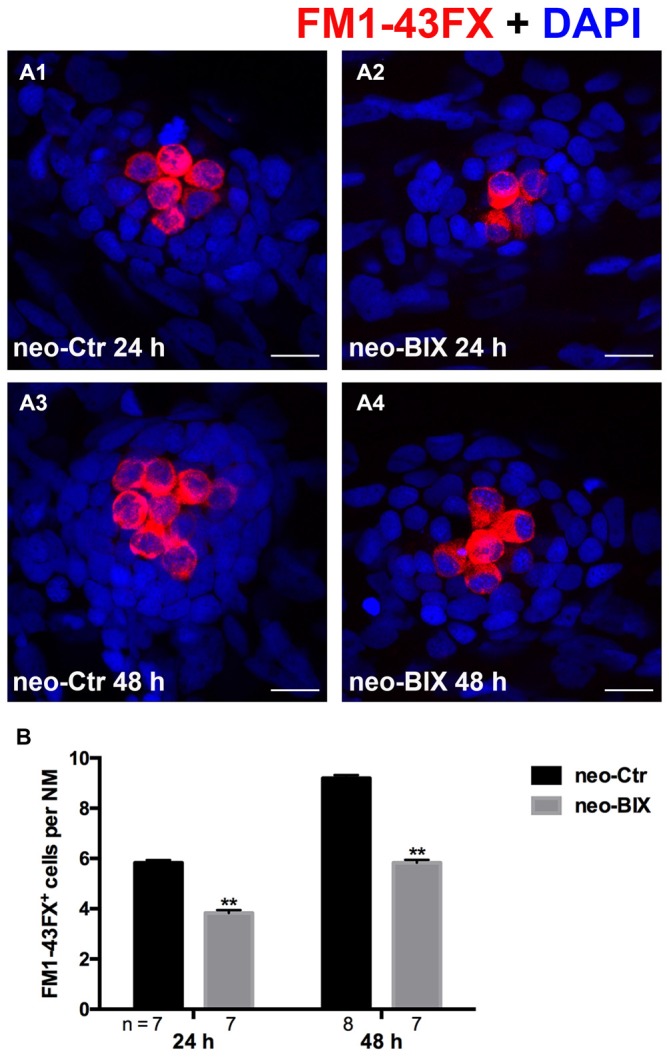
**BIX01294 treatment reduces FM1-43FX^+^ cells in the course of regeneration. (A)** The functional HCs of 5 dpf wild-type larvae are stained with FM1-43FX (red) with or without exposure to BIX01294 for 24 h or 48 h after neomycin damage for 1 h. Nuclei are stained with DAPI (blue). Scale bars = 10 μm. **(B)** The average number of FM1-43FX^+^ cells per NM in larvae treated with or without 20 μM BIX01294 for 24 h or 48 h after neomycin damage. Bars are mean ± SEM, and *n* = total number of embryos. **p < 0.001.

To test the functionality of the newly regenerated HCs, the wild-type larvae were stained with the vital dye FM1-43FX, which is a marker of functional mechanotransduction channels in HCs. We found significantly fewer FM1-43FX-positive HCs in the BIX01294-treated group than in controls at both 24 h and 48 h after 1 h neomycin treatment (Figures 3A,B), which is consistent with our findings in Tg(brn3c:mGFP) zebrafish and Myosin-VI staining in wild-type zebrafish.

**Figure 4:**
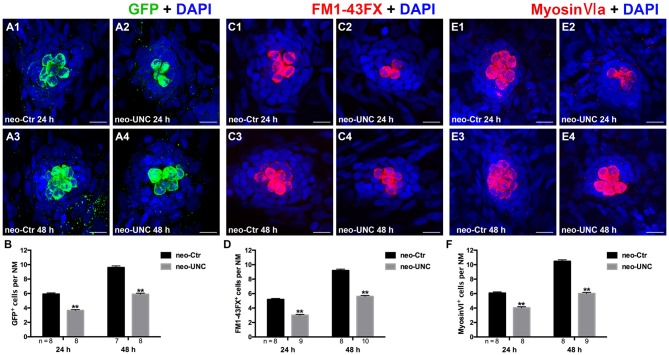
**Inhibition of G9a/GLP with UNC0638 also decreases the regeneration of HCs in lateral line NMs. (A)** We treated 5 dpf Tg (brn3c:mGFP) zebrafish with neomycin for 1 h and then treated them for 24 h or 48 h with 10 μM UNC0638. HCs in these fish are labeled with anti-GFP antibody (green), and nuclei are stained with DAPI (blue). Scale bars = 10 μm. **(B)** The average number of GFP^+^ cells per NM in larvae treated with or without 10 μM UNC0638 for 24 h or 48 h after neomycin damage for 1 h. Bars are mean ± SEM, and *n* = total number of embryos. **p < 0.001. **(C)** The functional HCs are stained with FM1-43FX (red). Nuclei are stained with DAPI (blue). Scale bars = 10 μm. **(D)** The average number of FM1-43FX^+^ cells per NM in larvae treated with or without 10 μM UNC0638 for 24 h or 48 h after neomycin damage. Bars are mean ± SEM, and *n* = total number of embryos. **p < 0.001. **(E)** HCs in the lateral line NMs were stained with Myosin-VI (red). Nuclei are stained with DAPI. Scale bars = 10 μm. **(F)** The average number of Myosin-VI^+^ cells per NM in larvae treated with or without 10 μM UNC0638 for 24 h or 48 h after neomycin damage for 1 h. Bars are mean ± SEM, and *n* = total number of embryos. **p < 0.001.

To verify our results and to eliminate the possibility of off-target effects of BIX01294, we treated the 5 dpf neomycin-damaged Tg(brn3c:mGFP) line and the wild-type larvae with another potent and selective G9a/GLP inhibitor UNC0638 for 24 h and 48 h. Similar decreases in HCs were detected by immunostaining of anti-GFP, FM1-43FX, and anti-Myosin-VI in the 10 μM UNC0638 treatment groups compared to corresponding parallel DMSO controls, and this confirmed that inhibition of G9a/GLP with BIX01294 and UNC0638 reduces HC regeneration after neomycin damage in the zebrafish lateral line neuromasts (Figures 4A–F).

### BIX01294 Treatment Inhibits Cell Proliferation and Induces Fewer SCs in Neuromasts

**Figure 5:**
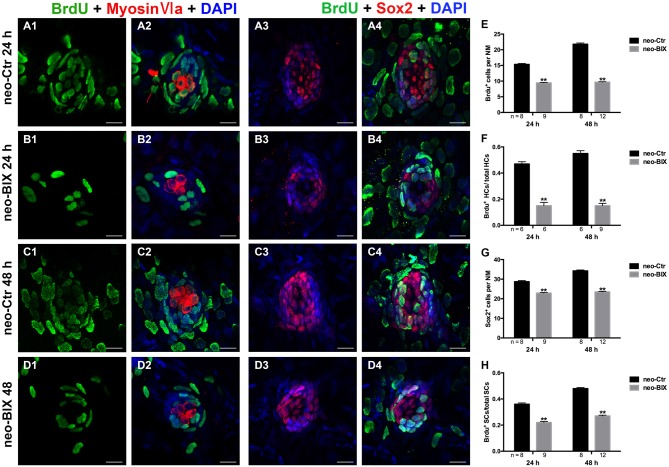
**BIX01294 significantly suppresses cell proliferation and induces reduced supporting cell (SC) production.** We treated larvae at 5 dpf with neomycin and monitored cell proliferation with or without BIX01294 over the next 2 days. **(A1–D2)** The BrdU antibody shows dividing cells (green) in the NMs of the zebrafish lateral line, and HCs are stained with Myosin-VI (red). Scale bars = 10 μm. **(A3–D4)** Lateral line SCs are stained with Sox2 antibody (red), and nuclei are stained with DAPI (blue). The BrdU antibody shows dividing cells (green) in the NMs of zebrafish. Scale bars = 10 μm. **(E)** BrdU^+^ cells were counted in control and BIX01294-treated larvae at 24 h and 48 h after neomycin damage for 1 h. **(F)** Quantification of the ratio of BrdU^+^ HCs in control and inhibitor-treated larvae at 24 h and 48 h after neomycin incubation for 1 h. **(G,H)** The number of SCs and quantification of the ratio of BrdU^+^ SCs in control and BIX01294-treated larvae at 24 h and 48 h after neomycin incubation for 1 h. Bars are mean ± SD, and *n* = total number of embryos. **p < 0.001.

In the zebrafish lateral line, 400 μM neomycin exposure for 1 h killed almost all of the mature HCs, and this was followed by the rapid regeneration of HCs (Harris et al., 2003). During regeneration, most of the replacement HCs were regenerated from proliferative cells labeled with BrdU (Ma et al., 2008). To further explore whether BIX01294 treatment affects cell proliferation during HC regeneration, we counted the number of BrdU^+^ cells in L1–L5 neuromasts at 24 h and 48 h following 400 μM neomycin treatment. As shown in Figures 5A1–B1,E, exposure to 20 μM BIX01294 for 24 h led to significant decreases in the number of BrdU^+^ cells per NM compared to controls (the control larvae harbored 15.35 ± 0.260 BrdU^+^ cells/NM and the BIX01294-treated larvae harbored 9.42 ± 0.178 BrdU^+^ cells/NM, *p* < 0.001). In the 48 h treatment, there were even greater decreases in BrdU^+^ cells per NM (control larvae harbored 21.78 ± 0.383 BrdU^+^ cells/NM and the BIX01294-treated larvae harbored 9.65 ± 0.161 BrdU^+^ cells/NM, *p* < 0.001; Figures 5C1–D1,E). Thus, BIX01294 significantly inhibited the proliferation of NM cells after neomycin-induced HC loss.

To investigate the mitotic regeneration of HCs, we co-stained the larvae with Myosin-VI and BrdU antibodies. We found fewer Myosin-VI/BrdU double-positive cells at 24 h in the BIX01294-treated neuromasts compared with the controls (Figures 5A2–B2). Furthermore, when comparing the ratio of Myosin-VI/BrdU double-positive cells to total HCs, there was a significant difference between the control and BIX01294-treated groups (the ratio in the controls was 0.47 ± 0.017, and in BIX01294-treated groups the ratio was 0.15 ± 0.026, *p* < 0.001; Figure 5F). At 48 h, the effect was even greater (the ratio in the controls was 0.55 ± 0.021, and in BIX01294-treated groups the ratio was 0.15 ± 0.018, *p* < 0.001; Figures 5C2–D2,F). These findings suggest that BIX01294 significantly decreases the mitotic regeneration of HCs after neomycin-induced HC loss in zebrafish neuromasts.

Each NM contains HCs and SCs, and the proliferation of SCs enables the replacement of HCs after damage. Thus, we next evaluated the effect of BIX01294 treatment on SCs after neomycin exposure. Sox2 is highly expressed in the SCs of zebrafish neuromasts, and to quantify the number of SCs we stained the larvae with a Sox2 antibody and counted the Sox2^+^ SCs in L1–L5 neuromasts of 8–12 fish at 24 h and 48 h post-treatment. We found significant reductions in the number of Sox2^+^ SCs at both time points after BIX01294 treatment (control larvae harbored 28.75 ± 0.545 and 34.25 ± 0.434 Sox2^+^ SCs per NM at 24 h and 48 h, respectively, and BIX01294-treated larvae harbored 22.87 ± 0.303 and 23.52 ± 0.248 Sox2^+^ SCs per NM at 24 h and 48 h, respectively, *p* < 0.001; Figures 5A3–D3,G).

Subsequent co-staining with Sox2 and BrdU antibodies was performed to detect the proliferation rates of NM SCs after neomycin damage at 24 h and 48 h. Consistent with the results described above for the mitotic regeneration of HCs, we found significant decreases in the ratio of Sox2/BrdU double-positive cells to total SCs after BIX01294 treatment at both 24 h and 48 h (the ratio in the controls was 0.36 ± 0.010 and 0.48 ± 0.008 at 24 h and 48 h, respectively, and in BIX01294-treated groups the ratio was 0.22 ± 0.008 and 0.27 ± 0.006 at 24 h and 48 h, respectively, *p* < 0.001). As the major source of newly regenerated HCs after neomycin damage, SCs were significantly affected by BIX01294 treatment, which could cause a further decrease in HC regeneration (Figures 5A4–D4,H).

### Effects of BIX01294 Treatment on Apoptosis

Our previous research confirmed the protective effect of BIX01294 on apoptosis from aminoglycoside-induced HC loss (Yu et al., 2013), suggesting that the decreased HC regeneration with BIX01294 might be mainly due to the inhibition of cell proliferation but not to increased cell death. To confirm our hypothesis, we used an antibody against cleaved caspase-3 to detect cell death during the HC regeneration process. We failed to find cells that labeled with cleaved caspase-3 both in the BIX01294 treatment groups and the controls in the zebrafish lateral line neuromasts (Supplementary Figure 1). Moreover, we failed to observe any significant difference in the apoptosis index (the number of apoptosis bodies/total number of DAPI cells) between the BIX01294-treated group and the control group. These results suggest that BIX01294 did not increase cell death in the lateral line neuromasts of zebrafish compared to the controls and that the inhibition of cell proliferation, but not apoptosis, was the main cause of decreased HCs in the course of regeneration.

### BIX01294 Treatment Down-Regulates the Wnt and Fgf Signaling Pathways

**Figure 6:**
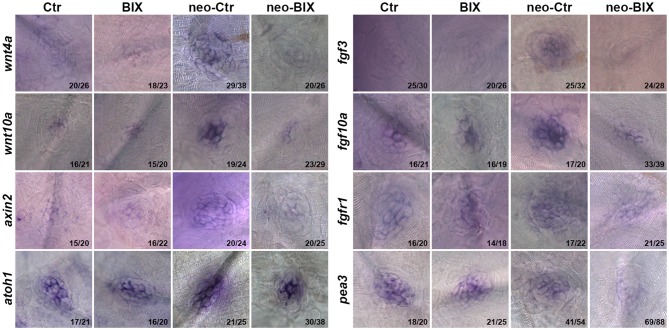
**BIX01294 significantly down-regulates the Wnt and Fgf signaling pathways.** Localization of markers of Wnt (*wnt4a*, *wnt10a*, and *axin2*) and Fgf signaling (*fgf3*, *fgf10a*, *fgfr1*, *pea3*, and *atoh1a*) with whole mount *in situ* hybridization in 6 dpf control embryos and BIX01294-treated embryos with or without neomycin treatment for 1 h. Expression of *wnt4a*, *wnt10a*, *axin2*, *fgf3*, *fgf10a*, *fgfr1*, *pea3*, and *atoh1a* was significantly down-regulated during the neomycin-induced regeneration period after BIX01294 treatment for 24 h. The ratios of representative images to total images were labeled, respectively.

Wnt and Fgf signaling play important roles in the development and regeneration of HCs in the zebrafish lateral line (Wright and Mansour, 2003; Munnamalai and Fekete, 2013). In this study, we investigated whether BIX01294 treatment reduced HC regeneration by disrupting the regulation of Wnt and/or Fgf signaling in the NM. At 24 h after neomycin treatment, the mRNA levels of *wnt4a*, *wnt10a*, *fgf10a*, and *fgf3* were significantly up-regulated compared with the non-neomycin treatment group, indicating that the Wnt and Fgf pathways were activated in the control larvae. In the two groups without neomycin-induced damage, the mRNA levels of *wnt4a*, *wnt10a*, *fgf10a*, and *fgf3* were of not significantly different between the control and BIX01294-treated larvae, suggesting that BIX01294 had no effect on the mature HCs. We found a striking down-regulation of Wnt ligand (wnt4a and wnt10a) and Fgf ligand (fgf3 and fgf10a) expression after exposure to 20 μM BIX01294 for 24 h after neomycin treatment compared with the neomycin-treated controls (Figure 6, Supplementary Figure 2). We next investigated the expression of representative downstream targets of Wnt and Fgf signaling in the NM. We also found a significant decrease in the expression of the Wnt signaling downstream target gene *axin2* in BIX01294-treated neuromasts after neomycin damage, and the expression of the Fgf signaling-dependent gene *pea3* was also strongly reduced in BIX01294-treated neuromasts compared with the controls after neomycin treatment. We examined the expression of the Fgf receptor fgfr1 in the regenerated neuromasts and found a similar down-regulation of fgfr1 expression in the BIX01294-treated group after neomycin damage. Furthermore, *in situ* hybridization data showed that the mRNA expression of *atoh1a*, a HC marker, was also significantly reduced in the BIX01294-treated groups after neomycin exposure (Figure 6). Taken together, these results suggest that BIX01294 treatment significantly down-regulated the Wnt and Fgf signaling pathways, which could lead to the inhibition of cell proliferation and HC regeneration after neomycin-induced HC loss in the zebrafish lateral line.

### Overexpressing the Effectors of Wnt and Fgf Signaling Pathways Can Rescue Phenotypes of Decreased HCs

To confirm our hypothesis that the decreased HCs in the course of regeneration were mainly due to the down-regulation of the Wnt and Fgf signaling pathways, we increased Wnt and FGF signaling with the addition of 6-bromoindirubin-3-oxime (BIO; 1 μM) and bFGF (2 ng/ml) to the co-incubations with DMSO or BIX01294 for 48 h following 1 h neomycin treatment.

**Figure 7:**
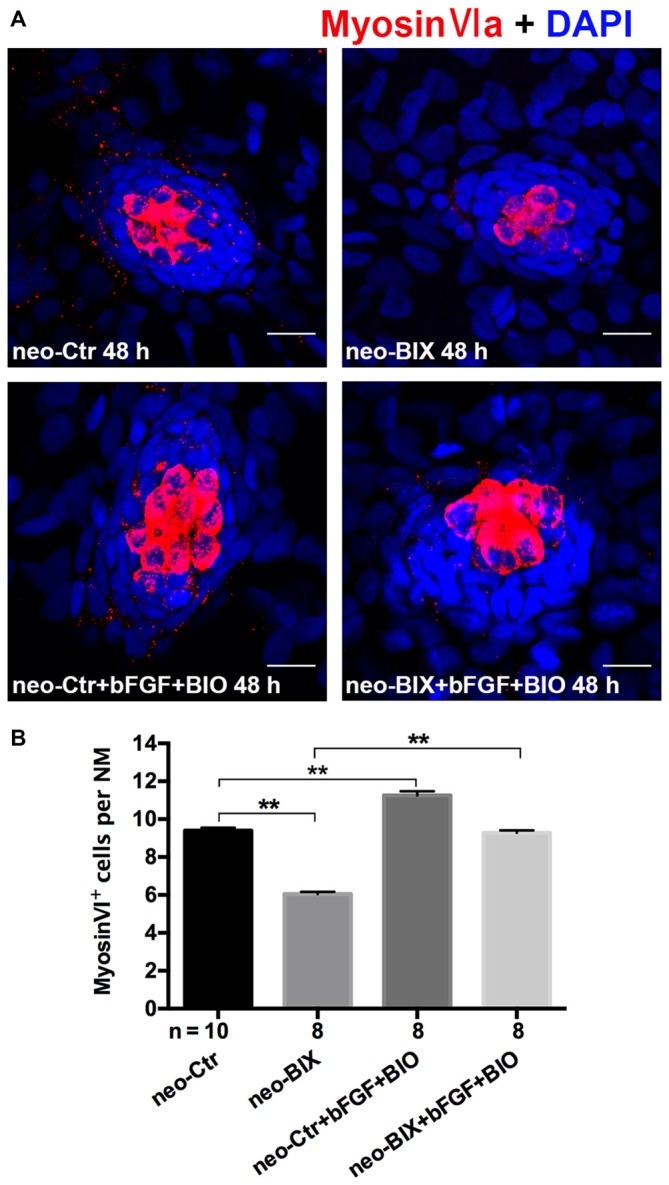
**The decreased HC regeneration after BIX01294 treatment following neomycin damage can be recued with the addition of bFGF and OF 6-bromoindirubin-3-oxime (BIO). (A)** Co-treatment of bFGF and BIO with BIX01294 after neomycin damage increases the number of HCs (Myosin-VI^+^, red) compared to the BIX01294-only treatment. Nuclei are stained with DAPI. Scale bars = 10 μm. **(B)** The average number of Myosin-VI^+^ cells per NM in larvae treated with or without BIX01294 for 48 h after neomycin damage for 1 h and the average number of Myosin-VI^+^ cells per NM after the addition of bFGF and BIO to controls or BIX01294-treated larvae. Bars are mean ± SEM, and *n* = total number of embryos. **p < 0.001.

We found a significant increase in the number of HCs by staining of Myosin-VI after the addition of bFGF and BIO compared to BIX01294 treatment zebrafish (the mean number of HCs in the neuromasts of the neomycin + BIX01294 + bFGF + BIO group was 9.28 ± 0.124 compared to 6.05 ± 0.119 in the BIX01294-only treatment group, *p* < 0.001). The addition of bFGF and BIO also increased HCs in the DMSO control (the control larvae harbored 9.40 ± 0.149 Myosin-VI^+^ HCs per NM, and the neomycin + bFGF + BIO treatment group harbored 11.25 ± 0.220 HCs per NM, *p* < 0.001). There was no significant difference in the number of HCs per NM between the neomycin + BIX01294 + bFGF + BIO group and the controls (*p* > 0.05; Figures 7A,B). These data together suggest that BIX01294 treatment decreased HCs mainly due to down-regulating the Wnt and Fgf signaling pathways, which can be rescued by the addition of the effectors of the Wnt and Fgf signaling pathways.

## Discussion

Epigenetic modifications have recently been reported to play important roles in cell development, survival, proliferation, and regeneration (Weaver et al., 2004; Stewart et al., 2009; Suvà et al., 2013; Roidl and Hacker, 2014). Previous studies have reported that the regulation of histone demethylation and methylation affects the regenerative ability in zebrafish fins (Stewart et al., 2009), and it has also been shown that histone deacetylases (HDAC) are required for zebrafish HC regeneration (He et al., 2014). This epigenetic modulation plays a dual role in gene activation and repression, and H3K9ac and H3K4me2 are markers of gene activation while H3K9me2 is known as a marker of gene repression (Felisbino et al., 2016). The lysine-specific demethylase 1 (LSD1) inhibitor trans-2-phenylcyclopropylamine (2-PCPA) increases the expression of H3K4me2, but its effect on H3K9me2 remains unclear. Our previous studies have reported that up-regulation of the acetylation signal and H3K4me2 level reduces HC regeneration in the lateral line neuromasts of zebrafish (He et al., 2014, 2016). Because treatment with the HDAC inhibitors valproic acid (VPA) and trichostatin A (TSA) significantly increase histone acetylation and decrease H3K9me2 levels (Felisbino et al., 2016), we hypothesized that the inhibition of H3K9me2 would have a similar impact on HC regeneration. In this study, we identified a novel role for histone dimethylase activity during lateral line HC regeneration by showing that pharmacological inhibition of G9a/GLP with BIX01294 significantly decreased the dimethylation of H3K9 in neuromasts. The loss of H3K9me2 significantly suppressed cell proliferation and reduced the number of SCs after neomycin exposure, and this led to reduced mitotic regeneration of HCs in the zebrafish lateral line. The decrease in HCs was verified using UNC0638, another potent G9a/GLP inhibitor. Furthermore, our *in situ* hybridization data demonstrated that loss of H3K9me2 significantly inhibited the Wnt/β-catenin and Fgf signaling pathways, and this suggests that the underlying mechanism behind the effects of BIX01294 on cell proliferation and HC regeneration might be through the inactivation of both the Wnt/β-catenin and Fgf signaling pathways. In addition, the reduced HC regeneration can be rescued by overexpressing the effectors of Wnt and FGF signaling pathways using BIO and bFGF. Therefore, our study suggests that epigenetic regulators might be a novel strategy for HC regeneration.

H3K9me2 is one of the most abundant and dynamic histone modifications, and the H3K9me2 pattern varies significantly during development and in disease pathogenesis (Bhaumik et al., 2007). Previous studies reported that H3K9me2 levels increase significantly during embryonic stem cell differentiation and that they decrease before the reprogramming of somatic cells to induced pluripotent stem (iPS), and this indicates a role for H3K9me2 in cell differentiation and cell fate determination (Lin and Dent, 2006; Li et al., 2011). However, the role of H3K9me2 in regulating cell proliferation and regeneration in the zebrafish lateral line remains unknown. In this study, we found that inhibition of H3K9me2 with the pharmacological G9a/GLP inhibitor BIX01294 significantly reduced the proliferation of SCs, which subsequently led to decreased numbers of SCs in the neuromasts. Multiple studies have demonstrated that SCs serve as the major source for regenerating new HCs after damage either through mitotic regeneration (Corwin and Cotanche, 1988; Fekete et al., 1998) or direct trans-differentiation (Roberson et al., 2004). Thus the inhibition of SC proliferation could be the cause behind the reduced HC regeneration seen after neomycin-induced HC loss. Indeed, our data clearly showed that down-regulation of H3K9me2 levels significantly reduced the number of mitotically regenerated HCs as well as the total number of HCs after neomycin exposure.

A previous study reported that the enrichment of H3K9me2 and G9a facilitate Wnt10a expression, thus activating the Wnt signaling pathway (Wang et al., 2013). Conversely, the down-regulation of H3K9me2 with a specific G9a inhibitor, UNC0638, inhibits the Wnt pathway (Kim et al., 2013). Thus we hypothesized that inhibition of H3K9 dimethylation with the G9a inhibitor BIX01294 would also down-regulate Wnt signaling in the zebrafish lateral line. In the zebrafish lateral line, Wnt activation is sufficient to maintain SCs in a proliferative state and to induce quiescent SCs to re-enter cell cycle, which leads to an increase in the number of HCs within both developing and regenerating neuromasts (Head et al., 2013). Conversely, Wnt inhibition suppresses this proliferation and reduces the HC number (Head et al., 2013; Jacques et al., 2014). In the mouse cochlea, it has been reported that Wnt activation in Sox2^+^ and Lgr5^+^ SCs results in proliferation and HC regeneration (Chai et al., 2012; Shi et al., 2013).

In this study, we performed *in situ* hybridization to detect the expression of key factors of the Wnt signaling pathway. We found that down-regulation of H3K9me2 with the G9a inhibitor BIX01294 significantly reduces the expression of the Wnt ligands *wnt4a* and *wnt10a* and that it inhibits expression of the Wnt signaling downstream target *axin2* in regenerating neuromasts. These findings suggest that down-regulation of H3K9me2 inactivates the Wnt signaling pathway and that this might subsequently inhibit SC proliferation and HC regeneration in zebrafish neuromasts. The detailed mechanisms through which H3K9me2 regulates Wnt signaling are worthy of further study.

In the zebrafish lateral line, Fgf signaling is required for primordium proliferation and migration as well as for NM deposition, and inactivation of the Fgf signaling pathway with the pharmacological Fgf receptor inhibitor SU5402 reduces the migration speed of the primordium and the rate of NM deposition along the body (Nechiporuk and Raible, 2008). Interactions between the Fgf and Wnt signaling pathways also play pivotal roles in regulating cell proliferation, regeneration, and migration in different cellular and biological contexts. Previous studies have shown that Wnt signaling plays an important role in regulating the Fgf pathway in the migrating primordium. Blocking Wnt/β-catenin signaling during primordium migration down-regulates the expression of *fgf3* and *fgf10*; conversely, activating Wnt/β-catenin signaling up-regulates genes in the Fgf signaling pathway (Aman and Piotrowski, 2008). However, the role of H3K9me2 in regulating Fgf signaling, and whether Fgf and Wnt signaling also interact during HC regeneration in zebrafish, have not been investigated yet.

In this study, we found that inhibition of H3K9me2 in regenerating neuromasts with the G9a inhibitor BIX01294 significantly decreased the expression of *fgf3* and *fgf10a*. We next addressed the requirement of Fgf signaling in HC regeneration by measuring *pea3* expression, which is a downstream target of the Fgf signaling pathway. We observed down-regulation of *pea3* in BIX01294-treated fish, which was consistent with the decreased *fgf3* and *fgf10a* expression and suggested that Fgf signaling in the regenerating NM had been inactivated. Taken together, these results demonstrated that BIX01294-induced inactivation of Fgf signaling might be another potential mechanism for disrupting SC proliferation and HC regeneration in the zebrafish NM.

In summary, we found that pharmacological inhibition of G9a/GLP with BIX01294 significantly down-regulated H3K9me2 levels and inhibited the proliferation of SCs and the regeneration of HCs after neomycin-induced HC loss in zebrafish lateral line neuromasts. The down-regulation of H3K9me2 also inactivated the Wnt and Fgf signaling pathways, and this disrupted SC proliferation and HC regeneration in neuromasts. Moreover, we confirmed our findings with the addition of BIO and bFGF to rescue the reduced HC regeneration. Our findings provide new insights into the mechanisms of HC regeneration in zebrafish and suggest that epigenetic regulation might be a novel therapeutic target for the treatment of hearing loss.

## Author Contributions

HL and QL designed the study, and made the decision to publish. DT performed the experiments. DT and YH co-worked in collecting and analyzing the data. DT and RC wrote and revised the manuscript.

## Conflict of Interest Statement

The authors declare that the research was conducted in the absence of any commercial or financial relationships that could be construed as a potential conflict of interest.

## Supplementary Material

Supplementary Figure 1 **Impact of BIX01294 treatment on apoptosis.** Cleaved caspase-3 staining (red) is used to label cell death. HCs are labeled with anti-GFP antibody (green), and nuclei are stained with DAPI (blue). Scale bars = 10 μm.

Supplementary Figure 2 **The representative images for fgf3, wnt4a, wnt10a, atoh1 and pea3**.
