# Supplementary figures and images for: Inhibition of H3K9me2 Reduces Hair Cell Regeneration after Hair Cell Loss in the Zebrafish Lateral Line by Down-Regulating the Wnt and Fgf Signaling Pathways
Source: Front Mol Neurosci. 2016 May 26;9:39. doi: 10.3389/fnmol.2016.00039 (PMC4880589; doi:10.3389/fnmol.2016.00039)

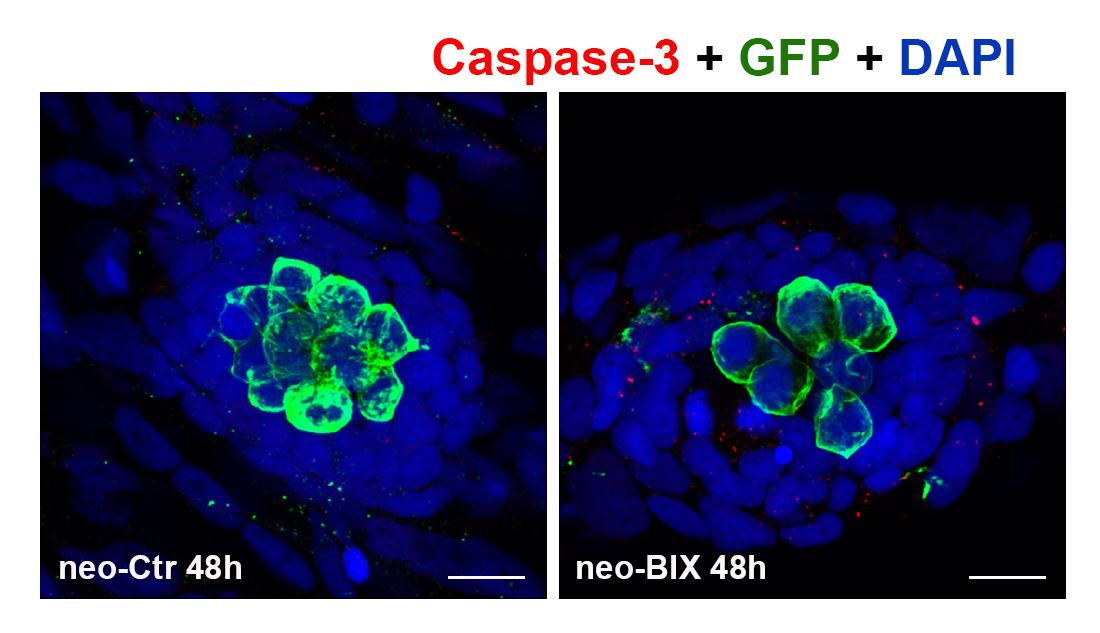

Supplement: Supplementary Figure 1 — Impact of BIX01294 treatment on apoptosis. Cleaved caspase-3 staining (red) is used to label cell death. HCs are labeled with anti-GFP antibody (green), and nuclei are stained with DAPI (blue). Scale bars = 10 μm. [file Image_1.tif]

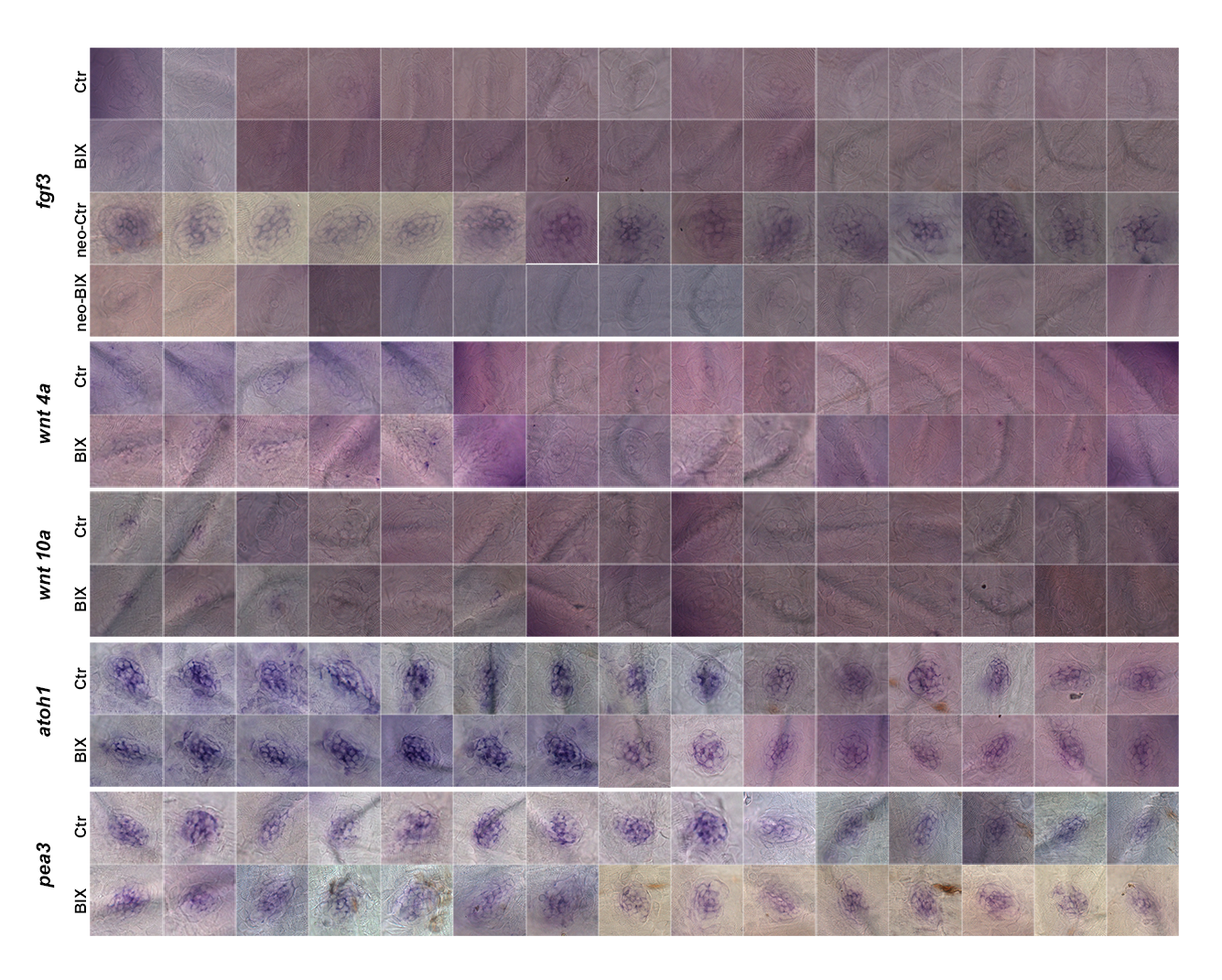

Supplement: Supplementary Figure 2 — The representative images for fgf3, wnt4a, wnt10a, atoh1 and pea3. [file Image_2.tif]
